# A Randomized Controlled Trial of a Blended Physical Literacy Intervention to Support Physical Activity and Health of Primary School Children

**DOI:** 10.1186/s40798-022-00448-5

**Published:** 2022-04-18

**Authors:** Ming Hui Li, James Rudd, Jia Yi Chow, Cindy Hui Ping Sit, Stephen Heung Sang Wong, Raymond Kim Wai Sum

**Affiliations:** 1 Department of Sports Science and Physical Education, The Chinese University of Hong Kong, Hong Kong, SAR China; 2 Department of Teacher Education and Outdoor Studies, Norwegian School of Sport Sciences, Oslo, Norway; 3 Physical Education and Sports Science, National Institute of Education, Nanyang Technological University, Singapore, Singapore

**Keywords:** Physical literacy, Sit–stand desk, Physical activity, Sleep, Cognitive function

## Abstract

**Background:**

The concept of physical literacy (PL) has been advocated as the need to create environments fostering sustainable engagement in PA. This study adopted ecological approach to evaluate the effectiveness of a blended PL intervention embedded into the school day to support children’s PA and health.

**Method:**

Designed as a three-arm randomized controlled trial, a total of 79 participants (59.5% girls) were randomly assigned to: the “Quantity + Quality” blended PL group combining sit–stand desks and play-based recess (SSPlay), the “Quality” group with play-based recess only (Play) or the control group. The intervention lasted for 13 weeks, and all the variables were collected at baseline, post-intervention and 3-month follow-up.

**Results:**

SSPlay and Play group significantly improved on two of the embodied PL domains, Physical Competence (− 2.96 vs − 5.15, *p* < 0.05) and Knowledge and Understanding (− 2.35 vs − 2.00, *p* < 0.05), total errors of cognitive flexibility (24.00 vs 12.92, *p* < 0.05), and this difference was maintained at follow-up (*p* < 0.05). Whilst there was no interaction effect between groups, and time effects were found for PA and planning from baseline to post-intervention.

**Conclusion:**

This was the first to adopt an ecological approach as an innovative strategy to provide the emergence of PA for children in Hong Kong. The blended intervention design that embedded both quantity and quality of PA into children’s school day has shown promise in supporting children’s all round development. PL intervention where environments are designed to increase the “Quantity + Quality” of children’s everyday interactions has led to improvements in PA and health outcomes, which may provide insights for future studies to adopt cost-friendly and feasible measures for promoting children’s PA in the school settings.

*Trial Registration*: ChiCTR, ChiCTR2000035038. Registered 29 July 2020—Retrospectively registered. http://www.chictr.org.cn/hvshowproject.aspx?id=46038.

**Supplementary Information:**

The online version contains supplementary material available at 10.1186/s40798-022-00448-5.

## Key Points

- This randomized controlled trial adopted Theory of Ecological Dynamics to inform the physical literacy intervention design by embedding quality and quantity of physical activity into the school day to support children’s health development in school settings.
- Effects in two domains of physical literacy and cognitive flexibility were found in the study population. There was no interaction effect between groups, time effects were found for physical activity and cognitive planning from baseline to post-intervention.
- Embedding both quantity and quality of PA into children’s school day has shown promise in supporting children’s all round development

## Introduction

The growing prevalence of leading sedentary lives among children and youth is an international concern, which is responsible for increased risk of childhood overweight and obesity, high blood pressure and mortality [1]. Research has shown that more than half of 6–11-year-old children do not meet the recommended levels of physical activity (PA) [2], and this unhealthy lifestyle acquired during childhood can be tracked into adolescents and adulthood [3]. In China, it has been shown that only 13.1% of children met the PA guideline set by the World Health Organization (2010) requiring at least 1 h of moderate-to-vigorous PA (MVPA) daily, while over 90% of school-aged children and youth have insufficient PA participation in Hong Kong [4]. Previous research has also reported that children in Hong Kong spent up to 32.3% of their waking time sitting, with their prolonged sitting time approximately 4.9 h/school day [5]. In response to the decreasing level of PA in children, schools have an important role for children to achieve the recommended PA levels and incorporating 24 Hour Movement Guidelines which require a comprehensive approach to support children’s increase in PA, reduce sedentary behaviour and improve sleep in Hong Kong [6].

Physical literacy (PL) has long been advocated as a framework which supports children’s ongoing engagement with PA within schools and educational context. At the heart of PL is the philosophy of phenomenology which takes the worldview that everyone brings a personal cluster of previous interactions to a situation, and each perceives the situation from a unique and personal point of view. Whitehead [7] did not, however, explain how this process of enrichment to support the development of PL occurs; the theory of Ecological Dynamics has been highlighted as a framework which can inform intervention design to support the enrichment of the quality and quantity of the movement experience [8]. For example, children in school move about their econiches (e.g. classroom, dinner hall, schoolyards, football pitches, basketball courts, aquatic environments etc.), and “information” (visual, acoustic, haptic and proprioceptive) specifies different environmental features which invite or repel (inter)action [9]. Ecological Dynamics, underpinned by both dynamical systems theory and ecological psychology, can explain the emergence of self-organized behaviour, common to all physical and neurobiological systems. It is the passage from one organized state of the system to another [8], and it allows an individual to navigate the environment, interact with others and negotiate tasks that support children achieve intended goals [10]. Thus, a child’s everyday interactions are a continuous cyclical process of exploration and revelation.

From an Ecological Dynamics perspective, a blended research design shifts the focus away from reductionist approaches [9]. Under this framework, the researcher becomes a landscape designer who supports the emergence of functional movement solutions to improve both the quantity and quality of participation in PA and reduce sedentary behaviour [11]. For example, a blended design could include manipulating environments to increase the quantity of PA such as incorporating sit–stand desks and also the adoption of more play time in different econiches and with different equipment supporting an enriched quality of PA [11]. To date, PL-directed interventions have focused on a top down and prescriptive approach to improve the teaching and learning experience to enhance children’s PA participation. Typically, these approaches are less learner centred and emphasize one common expectation of behaviour among learners. Importantly, these linear cause and effect models have shown small or even non-significant effect for PA or sedentary behaviour [12]. In contrast, approaches based on Ecological Dynamics (and will be termed as an ecological approach in this paper) take a systems approach that emphasizes that making small strategic changes to a child’s everyday environment is likely to have a nonlinear impact on increasing PA and reducing time for sedentary activity, which can lead to health benefits across all systems of the child [10]. Critically, an ecological approach promotes greater autonomy on the part of the students’ learning experience with the teacher or practitioner facilitating the learning process with a focus on learner-centeredness [13]. Therefore, the current study adopts a blended, ecological PL-oriented, “Quantity + Quality” design which increases the quantity of high quality play-based PA through environmental modifications in the classroom and wider school settings.

To the best of our knowledge, blended ecological PL interventions for improving the quantity and quality of PA have not been conducted in a school setting. An Ecological Dynamics blended intervention takes into consideration the confluence of sociocultural factors that are interacting to challenge the capacity of children in modern societies to lead an active lifestyle. Taking on the role of intervention landscape designer, the researcher looks to destabilize sedentary behaviours such as prolonged sitting at desks, thus incorporating sit–stand desks and increasing the quality and quantity of play-based recess across different school econiches. Together, this provides both a novel strategy to not only provide more PA but also to support wider health benefits such as cognitive function and health, thus promoting an all-round healthy lifestyle for students in Hong Kong primary schools. As PA has positive associations with physical, psychosocial and neurobiological development, it shows an increase for children’s motor and cognitive development, especially cognitive function [14]. In the Ecological Dynamics conceptualization, cognition plays an integral role in preparing and planning underpinning the intentionality, and “knowledge” of the modified environment [15]. Overall, the current study aimed to adopt an ecological approach to evaluate the effectiveness of a blended PL intervention embedded into the school day to support children’s PA and health. It was hypothesized that the blended “Quantity + Quality” PL group, compared with the single “Quality” group and the control group, will lead to significant improvement in objectively measured PA and PL, cognitive function in the domains of cognitive flexibility and planning.

## Methods

### Study Design

The detailed protocol for this pilot trial has been reported elsewhere [16]. As the theory of Ecological Dynamics was introduced post hoc for designing this study, it was not introduced in the protocol [16]. The efficacy of the ecological blended “Quantity + Quality” intervention was evaluated using a small-scale randomized controlled trial (RCT), with a three-arm design which focused on approximately 9-year-old (4th grade of primary school) students from one Hong Kong primary school, as previous research has reported a significant increase in sedentary behaviour in children aged 11 years and older [17]. This public school was located in the New Territories, Hong Kong, and according to the school report [18], all the 4^th^ grade participants’ families had a middle socioeconomic level. The study received ethics approval from Survey and Behavioural Research Ethics of the Chinese University of Hong Kong (Reference No. SBRE 18-108) and all the participants provided written parental consent forms prior to participation.

Randomization was conducted using Google random number generator to randomly assign the students to the intervention classes or control condition using a 1:1:1 ratio. Outcome data were collected at baseline, post-intervention and 3-month follow-up for all the variables. Baseline data collection was conducted before the intervention began in January 2019, the post-intervention measure for all the students was performed successively 13-week after the completion of the intervention during the end of semester in July 2019, and the follow-up measurement in all participants was performed 3 months after at the start of the next semester (October 2019). Reporting of the trial follows the CONSORT statement (Additional file 1).

### Participants and Intervention

**Fig. 1:**
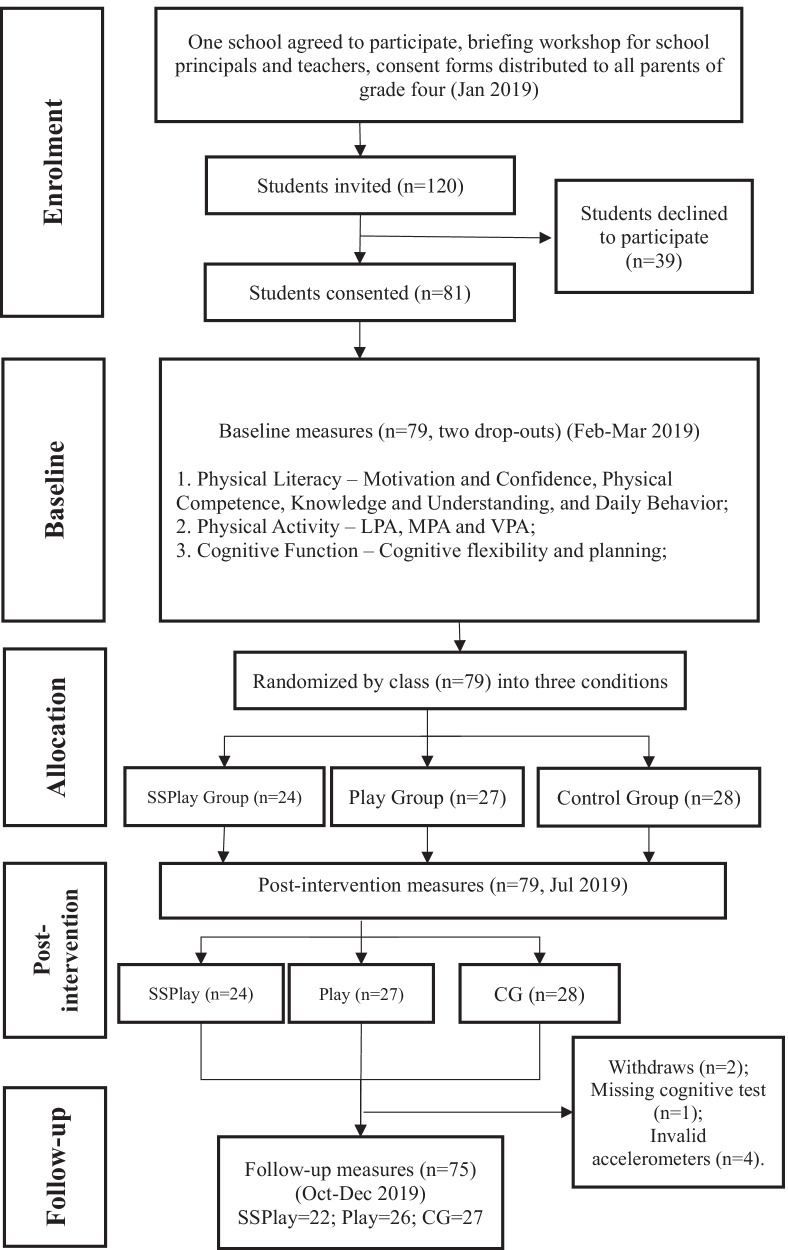
Flow of participants through the blended “Quantity + Quality” intervention

A sample size of 20 in each group (recruiting 24 with an assumed 20% attrition) would have at least power of 80%, an alpha of 0.05 and a moderate effect size (*f* = 0.25) [19] calculated by G-power software. All the grade four students (*n* = 133) were invited and distributed parent consent forms, and 81 students (response rate = 61%) agreed to participate and returned the forms. The children were excluded if they had a disability that prevented periods of standing or had an injury or illness that limited performing normal daily tasks. All the participants were randomly assigned to either of the three conditions (Fig. 1), which included a blended PL group (combining sit–stand desks and play-based recess; SSPlay), a single play-based group (Play), and a control group (CG).

Thirty height-adjustable sit–stand desks (Askisi 720, SMART Inc., USA) were placed in the SSPlay students’ classroom of the school for two academic semesters. Similar to Hinckson, Salmon [20]’s descriptions, the equipment could be moved up and down manually with the use of a lever and allowed the children to work in a seated or standing position. Prior to the intervention phase, a 3-h briefing session regarding the instructions on how to administer the sit–stand desks and PA-based recess was held for all the teachers and parents in order to support them in the development of classroom environment within schools. The research plan of breaking up prolonged sitting every 15 min during two regular classes (each class before the recess) per day could ensure all children in SSPlay group use the sit–stand desks for at least 1 h per day on average across the week [21]. Stools or chairs were retained in the classroom for them to feel free to choose whether they sat or stood when using the sit–stand desks. Teachers encouraged and led each child to stand when 15-min prolonged sitting was reached in the classroom (“Quantity”, e.g. both sit-to-stand transitions and stand-to-sit transitions). Besides, SSPlay and Play children also participated in a play activity during recess time, in which the unstructured outdoor interactive games were introduced to children and led by PE interns during recess (“Quality”, e.g. extra play-based opportunities). The extra play-based activities were up to 15 min in duration and twice a day across the week, which includes games such as skipping rope, shuttlecock kicking, hide-and-seek in the specific area, and supplemented with several minutes of cooling down. The students who were assigned to the control condition adhered to their regular class schedules and lesson delivery. They used their standard classroom desks in the classroom, with no experimental changes made to their classrooms.

### Measures

Children’s height and weight were measured by trained appraisers using standardized procedures, with children in light clothing and shoes removed, using TANITA measuring boards (Tanita RD-545-sv, Tokyo, Japan) and Seca (model 770 scales, Hamburg,

Germany). Body mass index (BMI, kg/m^2^) was then calculated from the measured weight (nearest 0.1 kg) and height (nearest 0.1 cm), with the standard equation (body weight [kg]/height [m]^2^).

PA was measured by ActiGraph GT3X + (Actigraph LLC, Pensacola, FL, USA), which were worn on the children’s waists for seven consecutive days. Monitors were initialized prior to data collection and were set to begin collecting data at the start of the school day on the Monday of every week. Data were collected in 10 s epochs to account for children’s natural activity levels, which generally occurred in short bouts [22] as it was shown to present the most acceptable classification accuracy for accelerometer use among children. Evenson cut-points (MVPA ≥ 2296 counts min − 1) were applied to intensity levels. As suggested by Aadland, Andersen [23], the non-wear period refers to a 45 or 60-min consecutive zero count-criterion in paediatric studies. The monitors had to be worn for at least 400 min/day for a minimum of 4 days, with at least one valid weekend included [24]. The accelerometers could be removed only during water activities, such as showering or swimming, and the participants had to fill in the information provided in the log sheets.

Physical literacy was measured using the Chinese version of the Canadian Assessment of Physical Literacy, second edition (CAPL-2, Chinese) [25], which was the first to comprehensively assess children’s PL in the Chinese context, comprising of four domains: Daily Behaviour (30 points); Physical Competence (30 points); Knowledge and Understanding (10 points); and Motivation and Confidence (30 points). The total achievable score for this assessment was 100 points. The whole CAPL-2 (Chinese) model was reported a good fit with construct validity: Chi-square (*χ*^2^ = 70.16, *df* = 43, *p* < 0.05), root mean square error of approximation (RMSEA) = 0.04, 90% CI (0.024–0.062), comparative fit index (CFI) = 0.94, Tucker–Lewis index (TLI) = 0.90, to be adopted to evaluate children’s PL [25].

Children’s aspects of cognitive function were assessed by two computer-based tasks, all of which were performed using the Inquisit 5 platform. Participants were required to perform the tasks in a quiet room under the supervision of an instructor who was trained prior to the testing. The classical version of the Wisconsin Card Sorting Test (WCST) with the standard number of 128 cards [26] was adopted to measure cognitive flexibility and working memory. This task consisted of 4 key cards and 128 response cards. Participants were instructed to sort the response cards, shown at the bottom of the screen, according to the characteristics of the key cards presented on the screen’s upper side, comprising the following categories: colours (red, green, yellow and blue); forms (triangle, star, cross and circle); and numbers [1–4]. The instructor was permitted to provide instructions relating to the categories either prior to or during the task, while feedback on “correct” or “incorrect” was presented after each selection. It took each participant approximately 20 min to complete the task. Both total and perseverative errors were recorded as target variables, since an increase in any of these variables indicated cognitive flexibility impairment [27]. The Tower of London Task, a widely administered neuropsychological assessment, was used for measuring the planning and problem-solving [28]. The task consisting of a practice trial and 12 test trials required the participants to move beans to solve problems. When presented with a graph on the screen showing three vertical pegs with graded heights and each holding beans (either 3, 2 or 1), the participants had to move the beans so as to be identical to the goal graph, without violating the rules [29]. It took each participant approximately 20 min to complete the task. Both the total correct and total move scores were derived for analysis, given that these variables were found to be influenced by sport-related exercises [29].

### Statistical Analysis

According to the CAPL-2 (Chinese) [25], missing raw scores in Physical Competence domain could be replaced using the recommended algorithm stated in the manual; therefore, the techniques were adopted to replace missing values for PL in the Physical Competence domain. For the measure of PA, individual information-centred approach was adopted for substituting missing data points [30]. This method was demonstrated as an effective method and superior to the group information-centred methods for handling accelerometer missing data when 7 days of data were collected. For each domain of PL, the missing values belonging to the Physical Competence domain and the Daily Behaviour domain could be calculated according to the fraction that the CAPL-2 (Chinese) manual provided. A maximum of one protocol could be completely missed and still have a calculated score [25].

Descriptive statistics were expressed as means and standard deviations for continuous variables, and gender was shown with percentage in male. All data were imported into SPSS version 23 for analysis. An *α* level of 0.05 was used for all statistical tests. Shapiro–Wilk and Levene tests were used to check the normality and homogeneity of data for both univariate and multivariate normality. Multivariate analysis of variance (MANOVA) test was used to assess between group comparisons at baseline, followed by the post hoc pairwise comparison with the Bonferroni adjustments. A two-factor mixed-design ANCOVA was conducted to assess the change in dependent variables over the 3 time points between groups, separately. Adjustments were made for sex, age and BMI category. Effect sizes (ESs) using partial eta squared were calculated and reported, large effect with *η*^2^ ≥ 0.14, medium effect with 0.14 > *η*^2^ ≥ 0.06, and *η*^2^ < 0.06 indicating small effects [31].

## Results

A final sample of 79 students (59.5% girls, mean age = 9.6 years [SD = 0.61, range 9.0–12.0]) were evaluated after completing the missing data. Due to several dropouts in the 3-month follow up test, 75 students were included for assessing PA and PL from baseline to follow-up (see Fig. 1). There were no significant differences (*p* > 0.05) between control and intervention groups at baseline for all measured variables. Descriptive statistics of baseline variables are presented in Table 1. Intervention effects of the blended “Quantity + Quality” intervention on all the outcomes over three measurement periods are shown in Table 2. Table 3 displays the mean changes in the measured variables from baseline to immediately post-intervention, while Table 4 displays that from baseline to 3-month follow-up.

**Table 1:** Baseline characteristics of study participants

	SSPlay (*n* = 24)	Play (*n* = 27)	CG (*n* = 28)	*p*
Age	9.7 ± 0.7	9.6 ± 0.6	9.6 ± 0.6	0.947
Male	9 (37.5%)	12 (44.4%)	11 (39.3%)	0.873
BMI (kg/m^2^)	16.8 ± 3.0	17.3 ± 3.1	16.9 ± 2.8	0.837
*Physical activity*				
Number of valid days	5.5 ± 1.5	5.4 ± 1.8	4.8 ± 1.9	0.262
LPA (min day^−1^)	201.6 ± 56.2	199.5 ± 39.3	204.1 ± 59.0	0.948
MPA (min day^−1^)	27.7 ± 9.3	24.5 ± 6.1	27.2 ± 10.2	0.361
VPA (min day^−1^)	11.4 ± 6.6	10.1 ± 3.0	12.8 ± 8.2	0.293
*Physical literacy*				
Motivation and confidence	22.8 ± 4.8	21.4 ± 5.0	22.0 ± 4.8	0.627
Physical Competence	14.7 ± 5.7	13.3 ± 3.5	14.6 ± 4.0	0.457
Knowledge and Understanding	4.5 ± 1.9	4.6 ± 1.3	4.0 ± 1.8	0.303
Daily Behaviour	10.5 ± 3.5	9.4 ± 3.2	10.8 ± 3.6	0.326
*Cognitive flexibility—WCST*				
Total errors	52.9 ± 18.0	51.4 ± 15.2	43.3 ± 19.2	0.120
Perseverative errors	8.3 ± 7.2	7.5 ± 2.4	6.9 ± 4.4	0.621
*Planning—ToL*				
Total correct	74.0 ± 12.7	73.3 ± 11.4	71.6 ± 14.4	0.800
Total move scores	29.0 ± 3.2	29.4 ± 2.5	27.8 ± 3.8	0.183

*BMI* body mass index, *LPA* light physical activity, *MPA* moderate physical activity, *VPA* vigorous physical activity, *WCST* Wisconsin card sorting test, *ToL* tower of London

**Table 2:** Characteristics and interventional interaction effects on primary outcomes; Mean ± SD 1

Outcome	Post-intervention			Group* time *p* value	Effect size	Follow-up			Group* time *p* value	Effect size
	SSPlay	Play	CG			SSPlay	Play	CG		
*Physical activity*										
LPA (min day^−1^)	216.3 ± 40.4	209.7 ± 42.8	195.8 ± 40.1	0.23	0.04	203.8 ± 42.9	191.2 ± 44.5	191.1 ± 31.7	0.70	0.01
MPA (min day^−1^)	32.7 ± 10.8	26.9 ± 7.2	26.7 ± 10.8	0.16	0.05	26.4 ± 10.1	22.9 ± 8.0	25.9 ± 8.3	0.85	0.004
VPA (min day^−1^)	13.8 ± 6.1	11.4 ± 3.8	11.9 ± 8.6	0.23	0.04	12.1 ± 8.0	9.2 ± 3.1	11.7 ± 7.2	0.45	0.02
*Physical literacy*										
Motivation and confidence	23.1 ± 5.1	22.0 ± 5.9	21.7 ± 4.8	0.83	0.01	22.7 ± 4.7	21.7 ± 4.8	22.6 ± 5.3	0.82	0.01
Physical Competence	17.9 ± 6.2	18.3 ± 4.3	17.0 ± 4.5	0.02*	0.11	17.4 ± 4.6	15.8 ± 4.0	17.0 ± 4.6	0.90	0.003
Knowledge and Understanding	6.8 ± 1.3	6.6 ± 1.9	6.2 ± 1.9	0.88	0.004	6.3 ± 1.5	6.0 ± 1.8	5.5 ± 2.0	0.82	0.01
Daily Behaviour	12.3 ± 5.2	12.3 ± 4.5	10.4 ± 3.9	0.004*	0.14	8.8 ± 3.3	8.5 ± 3.2	9.8 ± 4.5	0.77	0.01
*Cognitive flexibility—WCST*										
Total errors	28.9 ± 15.9	38.2 ± 19.1	30.2 ± 18.7	0.07	0.07	20.7 ± 14.3	29.8 ± 16.1	26.4 ± 17.4	0.71	0.01
Perseverative errors	5.5 ± 2.1	6.4 ± 2.3	7.1 ± 3.4	0.21	0.04	5.3 ± 1.8	6.0 ± 2.1	5.6 ± 2.1	0.44	0.02
*Planning—ToL*										
Total correct	76.2 ± 12.7	76.8 ± 11.0	81.3 ± 13.8	0.39	0.03	76.8 ± 14.4	81.9 ± 14.2	77.5 ± 15.3	0.80	0.01
Total move scores	30.0 ± 3.3	27.9 ± 3.9	26.1 ± 5.0	0.16	0.05	31.0 ± 3.9	29.5 ± 3.3	29.9 ± 4.0	0.23	0.04

*SD* standard deviation, *LPA* light physical activity, *MPA* moderate physical activity, *VPA* vigorous physical activity, *WCST* Wisconsin card sorting test, *ToL* tower of London

*p < 0.05 for time versus group interaction

**Table 3:** Adjusted mean changes (95% CI) in the outcomes from baseline to post-intervention (pre–post)

	SSPlay (*n* = 23)	Play (*n* = 25)	CG (*n* = 28)	ES (partial *η*^2^)
*Physical activity*				
LPA (min day^−1^)	− 14.67 (− 50.61, 21.27)	− 11.40 (− 32.49, 8.70)	8.23 (− 15.88, 32.33)	0.050
MPA (min day^−1^)	− 4.92 (− 12.57, 2.73)	− 2.32 (− 6.79, 2.15)	0.57 (− 2.83, 3.97)	0.051
VPA (min day^−1^)	− 2.49 (− 7.07, 2.09)	− 1.39 (− 3.56, 0.79)	0.91 (− 3.07, 4.89)	0.026
*Physical literacy*				
Motivation and confidence	− 0.33 (− 2.95, 2.29)	− 0.56 (− 3.47, 2.34)	0.30 (− 1.66, 2.26)	0.042
Physical Competence*	− **2.96 (**− **5.25, **− **0.67)**	− **5.15 (**− **6.85, **− **3.45)**	− **2.42 (**− **4.20, **− **0.63)**	0.101
Knowledge and Understanding*	− **2.35 (**− **3.57, **− **1.12)**	− **2.00 (**− **3.17, **− **0.83)**	− **2.25 (**− **3.22, **− **1.28)**	0.035
Daily Behaviour	− 1.39 (− 2.82, − 0.37)	− **3.00 (**− **4.17, **− **1.29)**	0.39 (− 0.56, 1.34)	0.033
*Cognitive flexibility—WCST*				
Total errors*	**24.00 (16.08, 31.92)**	**12.92 (4.19, 21.65)**	**13.00 (3.81, 22.19)**	0.043
Perseverative errors	**2.09 (0.75, 3.42)**	1.82 (− 1.71, 5.36)	− 0.40 (− 2.62, 1.82)	0.011
*Planning—ToL*				
Total correct	− 3.17 (− 13.29, 6.94)	− 4.68 (− 10.52, 1.16)	− **9.68 (**− **16.54, **− **2.82)**	0.013
Total move scores	− 0.87 (− 3.09, 1.35)	1.60 (− 0.13, 3.33)	1.71 (− 0.66, 4.09)	0.014

Bold values signifies *p* < 0.05

*ES* effect size, *LPA* light physical activity, *MVPA* moderate-to-vigorous physical activity, *RT* reaction time

*p < 0.05 for time (whole group)

**Table 4:** Adjusted mean changes (95% CI) in all the outcomes from baseline to follow-up (pre-follow-up)

	SSPlay (*n* = 23)	Play (*n* = 25)	CG (*n* = 28)	ES (partial *η*^2^)
*Physical activity*				
LPA (min day^−1^)	0.23 (− 12.19, 42.32)	7.11 (− 16.57, 30.79)	10.52 (− 17.83, 38.87)	0.050
MPA (min day^−1^)	0.79 (− 2.30, 3.88)	1.69 (− 2.33, 5.70)	1.89 (− 2.72, 6.50)	0.051
VPA (min day^−1^)	− 0.77 (− 4.98, 3.44)	0.88 (− 1.32, 3.08)	1.16 (− 1.23, 3.55)	0.026
*Physical literacy*				
Motivation and confidence	0.07 (− 2.39, 2.53)	− 0.28 (− 2.98, 2.41)	− 0.62 (− 2.61, 1.37)	0.042
Physical Competence*	− **2.67 (**− **4.81, **− **0.53)**	− **2.67 (**− **4.97, **− **0.37)**	− **2.35 (**− **4.31, **− **0.39)**	0.101
Knowledge and Understanding*	− **1.87 (**− **3.10, **− **0.64)**	− **1.32 (**− **2.28, **− **0.36)**	− **1.50 (**− **2.60, **− **0.40)**	0.035
Daily Behaviour	1.67 (− 1.58, 4.92)	0.00 (− 1.99, 1.99)	− 0.25 (− 5.76, 5.26)	0.033
*Cognitive flexibility—WCST*				
Total errors *	**22.78 (13.36, 32.21)**	**21.88 (14.15, 29.61)**	**26.54 (17.96, 35.11)**	0.043
Perseverative errors	**2.26 (0.82, 3.70)**	0.80 (− 1.21, 2.81)	2.64 (− 1.03, 6.31)	0.011
*Planning—ToL*				
Total correct	− 3.44 (− 14.75, 7.89)	− **9.12 (**− **16.89, **− **1.35)**	− 5.89 (− 13.41, 1.63)	0.013
Total move scores	− 1.96 (− 4.33, 0.41)	0.04 (− 1.51, 1.59)	− 2.04 (-4.09, 0.02)	0.014

Bold values signifies *p* < 0.05

*ES* effect size, *LPA* light physical activity, *MVPA* moderate-to-vigorous physical activity, *RT* reaction time

*p < 0.05 for time (whole group)

### Outcome Variables from Baseline to Post-intervention

Overall, significant Time × Group interaction effects were found for Physical Competence (*p* = 0.02) and Daily Behaviour (*p* = 0.004) during the period from baseline to post-intervention, while no significant interaction effect was found from baseline to follow-up. As shown in Table 2, although there was no significant interaction effect in PA, cognitive flexibility and planning, main time effects were found in all the outcome measures in SSPA group and PA group (except for planning in PA group) from baseline to post-intervention. The intervention groups of SSPlay and Play significantly increased scores of Physical Competence (SSPlay mean difference = 2.96, *p* < 0.05 and Play mean difference = 5.15, *p* < 0.05) and Knowledge and Understanding (SSPlay mean difference = 2.35, *p* < 0.05; Play mean difference = 2.00, *p* < 0.05), and CG also increased (Physical Competence = 2.42, *p* < 0.05; Knowledge and Understanding = 2.25, *p* < 0.05), respectively. Over post-intervention, it was observed that the CG experienced a slightly decline in Daily Behaviour (mean difference = 0.39, *p* > 0.05) and Motivation and Confidence (mean difference = 0.30, *p* > 0.05), but an increase was observed in two intervention groups. Particularly in the Play group, a significant increase was observed in the domain of Daily Behaviour (pre–post-test mean difference = − 3.00, *p* < 0.05).

The average measure for the SSPlay group regarding PA was significantly higher than the control group from baseline to post-intervention (Table 3), which supports the previous hypothesis. The LPA results show changes of 14.67 min day^−1^ for the SSPA intervention group and 11.40 min day^−1^ for the PA group from baseline to week 13 (mean difference: 3.27 min day^−1^, *p* = 0.84), and these results were also comparative for MPA (SSPA: 4.92 min day^−1^, PA: 2.32 min day^−1^, mean difference: 2.6 min day^−1^, *p* = 0.44) and VPA (SSPA: 2.49 min day^−1^, PA: 1.39 min day^−1^, mean difference: 1.1 min day^−1^, *p* = 0.58). Large effect size was found for Daily Behaviour between groups from baseline to post-intervention and medium effect size for Physical Competence between groups (*η*^2^ = 0.14; *η*^2^ = 0.11).

In terms of cognitive function, there was a time effect observed in the cognitive flexibility at post-intervention in all three groups (SSPlay group = 24.00 [95% CI 16.08, 31.92], *p* < 0.05; Play group = 12.92 [95% CI 4.19, 21.65], *p* < 0.05; and CG = 13.00 [95% CI 3.81, 22.19], *p* < 0.05), with no significant difference between groups. Improved performances of cognitive flexibility were also observed in perseverative errors, with SSPlay group reduced more (mean difference = 2.09, *p* < 0.05) at the post-intervention during this test.

### Outcome Variables from Baseline to 3-Month Follow-Up

From baseline to follow-up, all groups increased scores in Physical Competence (SSPlay mean difference = − 2.67, Play mean difference = − 2.67 and CG mean difference = − 2.35, all *p* < 0.05) and Knowledge and Understanding (SSPlay mean difference = − 1.87, Play mean difference = − 1.32 and CG mean difference = − 1.50, all *p* < 0.05, with no differences between them); neither significance in time effect nor interaction effect was observed in PA during this period. For cognitive function, there were no significant time × group interactions at post-intervention and follow-up time points. A time effect was observed in the cognitive flexibility trials at follow-up measures of all the three groups, with SSPlay mean difference = 22.78 [95% CI 13.36, 32.21], Play mean difference = 21.88 [95% CI 14.15, 29.61] and CG mean difference = 26.54 [95% CI 17.96, 35.11], all *p* < 0.05). Improved performances of cognitive flexibility were also observed in perseverative errors from baseline to follow-up, with SSPlay reduced significantly, suggesting that changes in perseverative errors from baseline to follow-up were greater (SSPlay mean difference = 2.26, *p* = 0.002) in the SSPlay group children compared to the control-group children. In addition, mean change of correct results in the planning tasks was significantly higher in the Play group (Play mean difference = − 9.12, *p* < 0.05) from baseline to follow-up, compared to other two groups (SSPlay mean difference = − 3.44, *p* < 0.05; CG mean difference = − 5.89, *p* < 0.05).

## Discussion

The aim of this RCT was to investigate the effects of a blended ecological intervention on children’s PA, PL and cognitive function. Based on the overall findings, the current study has shown significantly positive interaction effects on Physical Competence and Knowledge and Understanding subdomains of PL and main effects on PA and markers of cognitive flexibility, whilst in the domains related to planning, the blended design did not show any negative impact. The current evidence showed that the blended ecological intervention design, which modified the classroom unstructured play opportunities, may provide a promising and feasible strategy to reduce sedentary behaviour, improve children’s PA and benefit PL development that can foster a healthy lifestyle in children throughout their lifespan [7]. Although this was a small-scale RCT, this empirical work was the first to explore the use of an ecological dynamics perspective to design an intervention by improving the quantity and quality of PA in the school settings. It is assumed that skill acquisition through quantity and quality PA echoes with the on-going co-adaptation of an individual’s behaviour to dynamically changing, interacting constraints, individually perceived and encountered [9]. Therefore, the role of the practitioner is to design interventions that will create a landscape of affordance that will encourage the adaptation of varied and a diverse range of functional movement solutions which can lead to non-proportional changes to enhance children’s health.

Specifically, the intervention had a more positive influence on children’s Physical Competence and Daily Behaviour domains of PL in the SSPlay group from baseline to post-intervention compared to other groups, which partially supported our hypothesis regarding PL improvement. As the results showed that there was a continuous emergence of functional movement solutions which emerged from the blended ecological design and involve the repertoire of behaviours (cognition, perception and movements), it is aligned to the importance of developing PL and it can support each individual to progressively enhance performance in PA participation [8]. Furthermore, a play-based recess was regarded as the meaningful embodied movement experience within the school environment, which may combine the approach of incorporating activity during the school days. As such, children were able to develop their PL through unstructured play opportunities, which was consistent with some previous findings [32]. Such blended intervention design supplemented with teacher training can be effective to reduce youth sedentary behaviour and increase PA opportunities in classrooms and recess, in the absence of compensation effects outside of school time [32]. In addition, there was a statistically significant increase observed in the Knowledge and Understanding domain at both post-intervention and follow-up measures, with no time-group interactions displayed. This domain is measured through a self-reported questionnaire, and it is understandable that children might be curious about the right answer and would seek for it immediately before the next trial [25], which may influence the between-group effects.

Notably, there was a significant time effect on PA in the intervention groups over a 13-week period, with SSPlay group increasing more as hypothesized. The special focus of “Quality + Quantity” PA in the SSPlay group may explain these results, as it involves a nonlinear pedagogical approach and inherently generated more PA opportunities [11]. More freely chosen tasks could directly guarantee light-to-moderate intensity of PA in alignment with the playful pedagogy; especially blending the quality and quantity of PA would encourage children to rely on and build upon their own resources, and to provide own autonomy in such settings which students are centred and undoubtedly achieve higher autonomy compared to traditional learning contexts where teachers prescribe specific movement templates for students to rehearse, reproduce and adhere to [33]. The blended design did not account for the significance of VPA shown in the SSPlay and Play intervention groups, as SSPlay emphasized “Quantity” and “Quality” during the intervention, and it is possible that children spent more energy in the light-to-moderate PA opportunities generated through sit–stand desks, rather than through the possibility of having more VPA engagement during play-based recess. These aspects may well have influenced the results observed and should be considered when generalizing the findings. Despite this issue in delivering the blended ecological intervention, the RCT is still valuable, as for most of the outcomes, the two intervention groups had a comparable and significant effect. Recently, the Hong Kong government has launched the official strategic framework—*Towards 2025: Strategy and Action Plan to Prevent and Control Non-communicative Diseases (NCDs) in Hong Kong*, to prevent and control NCDs [34]. This has recommended that a 10% reduction in the number of insufficiently active youth and adults should be achieved by 2025. The current study could support this aim and provide insights for future intervention studies to be conducted in Hong Kong.

Another novel finding was the improvement in the children’s cognitive function. The results indicated that both intervention groups have shown significant reductions for total errors of cognitive flexibility, and SSPlay group improved more by reducing preservative errors, which offered partial support for our hypotheses. Such changes could be comparable with previous studies which were also conducted in the context of the use of sit–stand desks by children using a similar approach [35, 36]. One key distinction between this study and past studies lies in the absence of a blended ecological research design previously. Instead, the sit–stand desk intervention was only employed to explore the significant improvements in inhibitory control among 10–12 years old [35]. Besides, our current findings were also consistent with a study that adopted the WCST for evaluating cognitive flexibility when incorporating sit–stand desk intervention, in which the researchers found that the effect was marginally significant [36]. One possible explanation for these results may be the innovative structure of the ecological research design which blended both sit–stand desks and play-based recess for encouraging more PA opportunities for children within the school environment [11] rather than solely sit–stand desks or single play-based intervention. A systematic review has collectively summarized the evidence that exposure to more PA would be beneficial to children’s cognitive functioning because of the relationship between PA and the areas of the brain that support complex cognitive processes [37]. The result reflects that an ecological dynamics framework is needed and supported by participants and the dynamics of a performance environment [10, 15].

Despite the interesting findings, the results of this study have shown some limitations. First, only a small sample of participants were included as this was the first study that adopted a blended ecological research design in the school settings for promoting children’s PA and health behaviour. This may have been responsible for the null findings in several of the outcomes. Further research is recommended to include a larger sample of students to increase the generalizability of the results. Second, only Actigraph accelerometers were adopted to measure children’s PA, which were not as accurate as activPAL in measuring lower spectrum of PA. Despite between-monitor agreement in classifying sitting time under free-living conditions, activPAL provides a valid measure of posture; therefore, the results should be generalized with caution. Third, although the school reported that all families had a middle socioeconomic level, there may be some individual variabilities among participants that could confound the findings. Fourth, it should be noted that in this study BMI was the only indicator used to assess the nutritional status (instead of waist circumference or adiposity) of the participants, which should be regarded as one limitation. Last but not the least, although a full desk allocation system (a sit–stand desk for every child) guaranteed the optimal health benefits for the participants, only one school was recruited for this small-scale study as our intervention content was designed to be attentive with respect to location characteristics, such as cultural norms, the education system and prevailing teaching styles. Therefore, the present intervention should be interpreted with some caution regarding its generalizability of the trial findings.

## Conclusions

This pioneer study has adopted the blended ecological intervention design which took place in the classroom environment by incorporating sit–stand desks and play-based recess as the novel strategy. It has embedded with both quantity and quality of PA into children’s school day to encourage more PA opportunities for children to explore, perceive and experience their unique trajectory and development of skill acquisition. The blended “Quantity + Quality” intervention is feasible to improve children’s PL, PA and cognitive function through combining the measure of sit–stand desks during class hour and extra play opportunities during recess. This blended intervention may provide compelling evidence of adopting a cost-friendly and feasible measures with the direction of the teachers.

## Supplementary Information

**Additional file 1**. CONSORT 2010 checklist for RCT.

## Data Availability

The datasets generated and/or analysed during the current study are not publicly available but are available from the corresponding author on reasonable request.
